# Evaluation of the effect of cellular SMS reminders on consistency of antiretroviral therapy pharmacy pickups in HIV-infected adults in Botswana: a randomized controlled trial

**DOI:** 10.1080/21642850.2016.1271333

**Published:** 2017-01-09

**Authors:** Michael J. A. Reid, Andrew P. Steenhoff, James Thompson, Lesego Gabaitiri, Mark S. Cary, Katherine Steele, Susan Mayisela, Diana Dickinson, Peter Ehrenkranz, Harvey M. Friedman, Darren R. Linkin

**Affiliations:** a Division of Infectious Diseases, UCSF, San Francisco, CA, USA; b Botswana-UPenn Partnership, Gaborone, Botswana; c Perelman School of Medicine, University of Pennsylvania, Philadelphia, PA, USA; d Center for AIDS Research, University of Pennsylvania, Philadelphia, PA, USA; e Department of Pediatrics, Division of Infectious Diseases, The Children’s Hospital of Philadelphia, Philadelphia, PA, USA; f The Wharton School, University of Pennsylvania, Philadelphia, PA, USA; g Department of Medicine, School of Medicine, University of Botswana, Gaborone, Botswana; h Center for Clinical Epidemiology and Biostatistics, University of Pennsylvania, Philadelphia, PA, USA; i Independence Surgery, Gaborone, Botswana; j Bill & Melinda Gates Foundation, Seattle, WA, USA; k Department of Medicine, Division of Infectious Diseases, University of Pennsylvania, Philadelphia, PA, USA

**Keywords:** HIV, adherence, SMS, Botswana

## Abstract

**Objective:**

Several studies have demonstrated that cellular phone short message service (SMS) improve antiretroviral adherence for people living with HIV in Africa, although less data are available to support using SMS reminders to improve timeliness of antiretroviral therapy (ART) pharmacy pick up. This study tested the efficacy of SMS reminders on timeliness of ART pharmacy pickups at an urban clinic in Gaborone, Botswana.

**Design:**

A randomized-controlled trial evaluating the effect of SMS reminders on ART collection for patients with HIV on treatment.

**Methods:**

One hundred and eight treatment-experienced adult patients were enrolled and randomly assigned to a control group or an intervention group. Participants in the intervention group received SMS reminders that were sent in advance of monthly ART refills that needed to be collected. The primary outcome was 100% timeliness of pharmacy ART pickups. Secondary outcomes included frequency of physician visits, CD4 cell counts and viral loads.

**Results:**

Baseline characteristics in the intervention (*n* = 54) and control arms (*n* = 54) were similar. After six months, 85% of those receiving SMS reminders were 100% on time picking up monthly ART refills compared to 70% in the control group (*p* = 0.064). In secondary analysis, there were no significant changes in the CD4 counts and viral loads over the course of the study.

**Conclusions:**

Timeliness of ART pickup was not significantly improved by SMS reminders. Additionally, the intervention had no impact on immunologic or virologic outcomes in treatment-experienced patients.

## Introduction

Antiretroviral therapy (ART) has significantly reduced morbidity and mortality for people living with HIV/AIDS in Sub-Saharan Africa (SSA) (Palella et al., 1998). While initial concerns about poor adherence and widespread drug resistance have not been realized, evidence suggests that medication adherence among individuals in Sub-Saharan Africa does decline over time (Byakika-Tusiime et al., 2009; Mannheimer et al., 2005). Given the prohibitive costs of second and third line therapy, successful adherence support and retention interventions may be cost saving (Unge et al., 2009).

Mobile phone text messages using the short message service (SMS) have been demonstrated to be a feasible and affordable means of improving ART adherence (Finitsis, Pellowski, & Johnson, 2014; Kelly & Giordano, 2011; Mills et al., 2014; Pop-Eleches et al., 2011; Riaz, Riaz, Hussain, & Kherani, 2012; Vodopivec-Jamsek, de Jongh, Gurol-Urganci, Atun, & Car, 2012). Furthermore, mobile phones are widely available across Africa (Bank, 2012). We undertook a randomized-controlled trial at a single site in Gabor-one, Botswana, to assess whether SMS reminders improved timeliness of pharmacy pickup of antiretroviral medications for HIV-infected adults.

## Methods

From April 2008 to July 2009, we completed a randomized-controlled trial testing the efficacy of SMS texts to improve timeliness of pharmacy pickup visits among a sample of HIV-infected adults. The study was conducted at Independence Surgery, an urban private clinic in Gaborone, Botswana’s capital city. At the time of the study, all participants resided within cell phone network coverage.

Potentially eligible subjects were HIV-infected adults ≥21 years old on ART and receiving HIV care at the study clinic. Patients were ineligible if they did not intend to have continuous follow-up care and monthly medication refills at Independence Surgery for at least three months after enrollment (e.g. due to travel or studying abroad). Eligible patients were informed about the study and then asked to consent in either English or Setswana. Those individuals who did not already own a phone were provided with one for the duration of the study. The protocol was approved by the Institutional Review Boards of the Botswana Ministry of Health and the University of Pennsylvania and registered at www.clinicaltrials.gov, NCT01001741 prior to study commencement.

## Design

Participants were screened, consented, and enrolled by the study research staff on site. They were then randomly assigned in a 1:1 ratio to the intervention group (SMS reminder messages) or the control group (usual care). Randomization, laboratory assays, and analyses were done by investigators masked to treatment allocation. Study participants and clinic staff could not be masked because the intervention required overt participation.

## Intervention

The intervention, including phrasing of the SMS reminders, was developed after extensive consultation with clinic staff and expert patients. Those participants randomized to the intervention arm received SMS reminders three days prior, one day prior, and the morning of the scheduled monthly pharmacy pickup. All messages were fewer than 160 characters and did not specify HIV or ART in order to maintain confidentiality of HIV status. Messages were automatically sent out using the Independence Clinical Decision Support System (Wharton Entrepreneurial Programs, Philadelphia, 2006). The service sent ‘one-way’ messages to which recipients could not respond.

## Data collection and measures

Participants collected antiretroviral refills monthly, as per the clinic’s standard approach at that time. The primary outcome, timeliness of ART pharmacy pickups, was calculated from the number of attended pharmacy visits from 14 days after enrollment through 187 days after enrollment. A total of six or more visits during the six-month period was considered 100% adherence to pharmacy pickups. Fewer visits were divided by six to obtain a percentage of adherence. Thus, three visits equated to 50% adherence. The 100% level was chosen as the primary outcome because HIV regimens require greater than 95% adherence for maximal effectiveness and less than 100% ART pick up during the study period would have led to significantly less than 95% ART adherence. Secondary outcomes included suppression of plasma HIV-1 viral load (VL), defined as 400 copies per ml or less, CD4 counts, and the number of physician visits during the study period. Pharmacy records were recorded monthly by study staff in the clinic’s dispensary. Clinic visits for other reasons – such as to visit with a clinician – were not documented as evidence of ART pick up.

## Data analysis

Analyses were performed using an intention-to-treat approach. Baseline and outcome differences between the two groups were tested in bivariate analyses with parametric and nonparametric statistics, as appropriate. Logistic regression was used to determine if there was association between baseline characteristics and 100% adherence. We regressed the adherence on intervention, order of recruitment, and one baseline covariate at a time to find covariates with a significance of *p* < 0.10. Order of recruitment was included in regression analysis in order to mitigate for unanticipated recruitment bias. All analyses were conducted with SAS Version 9.3 (SAS Institute, Cary, N.C., USA). With a sample of 108, the study had >80% power to detect an improvement in baseline adherence of 67% to 90% of pharmacy pickup of antiretrovirals between the treatment and control arms.

## Results

### Sample characteristics

One hundred and eight participants were enrolled and randomized (*n* = 54 intervention, *n* = 54 control) and were included in all subsequent analyses. An additional 20 participants participated, in whom random allocation did not occur, were excluded from the study. Among the 108 participants, mean age was 40.8 years in the intervention arm, and 41.4 years in the control arm. More than 70% in both arms had been on ART for at least 4 years at the time of enrollment. There were no significant differences in demographic characteristics between intervention and control participants, including age, education, residence, and gender (Table 1). Cell phone ownership did not vary by treatment arm; 96% of all participants owned a phone at the time of enrollment. Groups were also balanced by distance from the clinic and type of ART regimen (Table 2).

## Intervention efficacy

In the post-intervention assessment, 85% of participants receiving SMS reminders demonstrated 100% six-month timely pharmacy pickups compared to 70% timely ART pickup in the control group (*p* = 0.064). Using logistic regression to predict 100% timeliness to pharmacy pickups, the odds ratio (95% confidence interval) for SMS reminders was 2.42 (0.94, 6.27). Including both the intervention and order of recruitment in the regression analysis produced an odds ratio of 2.40 (0.90, 6.42) for SMS reminders and 9.96 (1.72, 57.67) for the order of recruitment, where the first patient recruited was assigned a 1, the last a 0, and all those in-between an evenly spaced fractional value. In secondary analysis, there were no significant differences in the changes of CD4 count between the intervention and control groups. While mean log HIV VL was lower in the intervention group at the end of six months (5.31 vs. 3.88, *p* = 0.05), the difference in VL from baseline across the groups was not statistically significant (−0.24 vs. 0.09, *p* = 0.14). There was also no difference in frequency of clinic visits or loss to follow-up rates between the two groups. No adverse events occurred during the study period.

## Discussion

In this study, receiving SMS reminders did not lead to a significant improvement in timeliness of ART refill pick up. In addition, after six months of follow up, we found no evidence that the intervention had had a beneficial impact on clinical, immunologic, or virologic outcomes.

It is important to examine plausible factors that contributed to the intervention not producing the intended effects. First, it is possible that the short time frame of the RCT – six months of follow up – was inadequate to characterize a measurable effect. Secondly, we acknowledge that the intervention’s effect may have been mitigated by the type of patients enrolled; the majority (70%) had been on ART for at least 48 months at enrollment into the study. Furthermore, at baseline, most patients in both intervention and control arm had very low or undetectable viral loads (under 400 copies/ml), suggesting that many were already reasonably adherent with their medications. Such patients may be more accustomed to the routine of picking up medications each month. We speculate that the SMS reminder may be more effective in treatment-naïve populations, who are less likely to have established cues to help remind them to pickup medications each month and have the greatest challenges in establishing adherence habits (Wilson et al., 2013).

Notably, our negative results do not support the findings of other published trials in Kenya (Lester et al., 2010; Pop-Eleches et al., 2011) and Cameroon (Mbuagbaw et al., 2012) that have reported positive impacts of SMS reminders on medication adherence (Mbuagbaw et al., 2013). While interventions and outcomes in these studies were different, they demonstrated that text messaging can have significant effects on adherence to ART, although this effect was influenced by level of education, gender, timing (weekly vs. daily) and SMS interactivity.

To our knowledge, our study is the first to explore the role of SMS as a tool to improve pharmacy pickup in an African setting. One possible explanation for lack of significant finding in our analysis was that reminding people to pick up the pills is far less likely to impact pill adherence than interventions focused on improving real-time ART adherence. A number of recent studies (Haberer et al., 2016; Orrell et al., 2015; Sabin et al., 2015) have demonstrated the benefit of using SMS reminders in tandem with real-time electronic adherence monitoring devices that record when medications containers are opened and communicate the data to a central server, such as the Wisepill (“Wisepill Technologies,” 2016). However, recent evidence suggests that real-time electronic adherence monitoring and support is particularly beneficial for those initiating ART, compared to those with prior ART experience (Haberer et al., 2017). While our analysis failed to demonstrate a significant effect, our results build upon and confirms previous work indicating that effective SMS ART adherence interventions should be individualized to those most likely to benefit from them (Sabin et al., 2010; Simoni, Amico, Smith, & Nelson, 2010). Wireless monitoring and text reminder interventions are relatively simple and can be used as a tool by providers and adherence counselors already in the field. The potential for broad scalability may make it feasible to target specifically patients known to be poorly adherent or those who develop resistance (Sabin et al., 2010).

This RCT has several potential limitations. We acknowledge that our sample size was small and so we were limited in ability to detect a smaller difference in outcomes between the control and intervention groups. Furthermore, we excluded 20 study participants that were not randomly allocated to the intervention or control groups. Notably, ethnic or socioeconomic data on study participants was not documented. Furthermore, we acknowledge that the measure of retention to care used in this study (pharmacy pickups) may have over-estimated antiretroviral adherence rate. Nevertheless, timely pharmacy pickup is a commonly accepted metric of medication adherence (Liu et al., 2001; Martin et al., 2009) that correlated strongly with virologic suppression in our analysis and serves as a sensitive marker of loss to follow up in real-world practice (Orrell, Leisegang, Bangsberg, Maartens, & Wood, 2016). Finally, we acknowledge that the time from study completion to publication may limit the relevance of the findings, not least because of rapid scale up in use of SMS interventions to support HIV programs across SSA in the interim. However, we believe that our results remain relevant insofar as they illustrate the marginal benefit of SMS reminders to improve ART adherence in treatment-experienced patients who are doing well on therapy.

## Summary

SMS reminders did not significantly improve timeliness of ART pharmacy pickups among treatment-experienced patients accessing care at an urban private clinic in urban Botswana. Additionally, reminders had no impact on immunologic or virologic outcomes. SMS reminders might have a greater impact in other settings or with a less experienced patient population.

## Figures and Tables

**Table 1:** Analytic sample: baseline characteristics by randomization group.

No. (%)	No intervention (n = 54)	Intervention: text message (n = 54)
**Characteristic**		
Sex		
Male	29 (53.7)	31 (57.4)
Female	25 (46.3)	23 (42.6)
Age		
17–34	9 (16.7)	8 (14.8)
35–49	39 (72.2)	38 (70.4)
50–67	6 (11.1)	8 (14.8)
Years of post-primary education		
None	25 (46.3)	29 (53.7)
1–2	6 (11.1)	7 (13.0)
3 or more	23 (42.6)	18 (33.3)
Travel time to clinic		
Less than one hour	39 (72.2)	32 (59.3)
One hour or more	15 (27.8)	22 (40.7)
Pay any portion of medication costs?		
No	6 (11.1)	3 (5.7)
Yes	48 (88.9)	51 (94.4)
Does cost prevent regular treatment?		
No	52 (96.3)	52 (96.3)
Yes	2 (3.7)	2 (3.7)
Time of ART initiation:		
Four years ago or less	14 (25.9)	15 (27.8)
More than four years	40 (74.1)	39 (72.3)
Experience discomfort or side effects from medications?		
No	41 (75.9)	42 (77.8)
Yes	13 (24.1)	12 (22.2)
Do side effects make it difficult to take regular treatment?		
No	53 (98.2)	54 (100.0)
Yes	1 (1.9)	0 (0.0)
Disclosure of HIV status:		
No	6 (11.1)	8 (14.8)
Yes	48 (88.9)	46 (85.2)
Is your illness a secret from others in your family?		
No	19 (35.2)	21 (38.9)
Yes	35 (64.8)	33 (61.1)
Is your illness a secret from others in your community?		
No	5 (9.3)	5 (9.3)
Yes	49 (90.7)	49 (90.7)

**Table 2:** Analytic sample: outcome measures.

No. (%)	No intervention (n = 54)	Intervention: text message (n = 54)	P value
**Adherence**			
0 (no visits)	3 (5.6)	0 (0.0)	
17% (1 visit)	4 (7.4)	3 (5.6)	
33% (2 visits)	0 (0.0)	1 (1.9)	
50% (3 visits)	1 (1.9)	1 (1.9)	
67% (4 visits)	4 (7.4)	2 (3.7)	
83% (5 visits)	4 (7.4)	1 (1.9)	
100% (6 or more visits)	38 (70.4)	46 (85.2)	0.27
**Doctor visits**			
None	44 (81.5)	45 (83.3)	1.00
One	7 (13.0)	7 (13.0)	
Two	3 (5.6)	2 (3.7)	
**Laboratory results**			
Mean CD4, enrollment (SD)	378.1 (290.6)*	405.9 (243.3)§	0.62
Mean CD4, at last visit in past six months (SD)	389.5 (281.2)*	432.1 (269.0)§	
Mean Change in CD4 (SD)	9.2 (93.6)*	20.9 (88.7)§	0.53
Log of VL, at enrollment (SD)	5.59 (3.76)†	3.90 (1.89)††	0.005
Log of VL, at last visit in past six months (SD)	5.31 (3.60)†	3.88 (2.03)††	0.005
Change in Log VL	−0.24 (1.39)†	0.09 (0.53)††	0.14

* N = 48.

§ N = 47.

† N = 45.

†† N = 46.
